# 

**Published:** 1877-02-20

**Authors:** 


VOL. VII. FEBRUARY 20, 1877. No. 2.

PLEASE PRESENT THE CLAIMS OF THIS JOURNAL TO YOUR MEDICAL BRETHREN.

SHORT COMMUNICATIONS AND PRACTICAL ITEMS SOLICITED.

### THE SOUTHERN MEDICAL RECORD.

A MONTHLY JOURNAL OF PRACTICAL MEDICINE.

"*QUICQUID PRÆCIPIES ESTO BREVIS.*"

JAS. P. HARRISON & CO., PRINTERS.
ATLANTA, GEORGIA.

TERMS: $2.00 per Annum in Advance—Club Rates, 5 Copies for $8.00.

